# Traffic Lights Detection and Recognition Method Based on the Improved YOLOv4 Algorithm

**DOI:** 10.3390/s22010200

**Published:** 2021-12-28

**Authors:** Qingyan Wang, Qi Zhang, Xintao Liang, Yujing Wang, Changyue Zhou, Vladimir Ivanovich Mikulovich

**Affiliations:** 1 School of Measurement-Control and Communication Engineering, Harbin University of Science and Technology, Harbin 150080, China; wangqy@hrbust.edu.cn (Q.W.); zhangqi2020300093@stu.hrbust.edu.cn (Q.Z.); mirrorwyj@hrbust.edu.cn (Y.W.); 1820310200@stu.hrbust.edu.cn (C.Z.); 2 School of Radio Physics and Electronics, Belarusian State University, 220030 Minsk, Belarus; falcon@tut.by

**Keywords:** traffic light, object detection, YOLOv4, deep learning, computer vision

## Abstract

For facing of the problems caused by the YOLOv4 algorithm’s insensitivity to small objects and low detection precision in traffic light detection and recognition, the Improved YOLOv4 algorithm is investigated in the paper using the shallow feature enhancement mechanism and the bounding box uncertainty prediction mechanism. The shallow feature enhancement mechanism is used to extract features from the network and improve the network’s ability to locate small objects and color resolution by merging two shallow features at different stages with the high-level semantic features obtained after two rounds of upsampling. Uncertainty is introduced in the bounding box prediction mechanism to improve the reliability of the prediction of the bounding box by modeling the output coordinates of the prediction bounding box and adding the Gaussian model to calculate the uncertainty of the coordinate information. The LISA traffic light data set is used to perform detection and recognition experiments separately. The Improved YOLOv4 algorithm is shown to have a high effectiveness in enhancing the detection and recognition precision of traffic lights. In the detection experiment, the area under the PR curve value of the Improved YOLOv4 algorithm is found to be 97.58%, which represents an increase of 7.09% in comparison to the 90.49% score gained in the Vision for Intelligent Vehicles and Applications Challenge Competition. In the recognition experiment, the mean average precision of the Improved YOLOv4 algorithm is 82.15%, which is 2.86% higher than that of the original YOLOv4 algorithm. The Improved YOLOv4 algorithm shows remarkable advantages as a robust and practical method for use in the real-time detection and recognition of traffic signal lights.

## 1. Introduction

Traffic light detection technology can help drivers to identify the status of traffic lights and make decisions quickly according to the identified status of traffic lights. This can reduce driver distraction and prevent the occurrence of non-standard driving and illegal behavior. Therefore, this research on a traffic light detection and recognition model with a high accuracy in real-time has practical significance and broad development prospects for improving road traffic safety. Traffic light detection systems often use industrial cameras to collect road condition information. Due to the complexity and changeability of the traffic light image background in real traffic scenarios, the traffic light in an image occupies less pixels and its feature structure is sparse, which increases the difficulty of algorithm recognition. Therefore, it is very important to study a more effective small target detection algorithm for traffic signal light detection [1,2].

Traffic light detection has undergone several years of development, and many excellent detection methods have been reported. At present, these methods can mainly be divided into two categories [3]: one is based on traditional algorithms using image processing and machine learning, while the other is based on deep learning.

The use of traditional algorithms in traffic light detection has been widely investigated. In [4], the authors located the position of the traffic light by converting the color image to a YCbCr color space. The problem of low detection and recognition accuracy due to traffic lights being a small target and only using their color and shape information was solved by taking advantage of Gabor wavelets’ sensitivity to image edges. At the same time, the authors extracted the features using two-dimensional independent component analysis and completed the traffic light classification by sending the features to the nearest neighbor classifier. In [5], the authors converted an RGB image to LUV color space on the basis of sliding window detection, used the aggregate channel features (ACFs) algorithm for training, then completed the detection of traffic lights. The authors of [6] proposed a multi-scale traffic signal detection method based on the ACF algorithm and an enhanced tree classifier. This method used a feature pyramid to fuse features and was able to effectively detect red and green traffic lights. In [7], the authors proposed the use of a fast image segmentation and compression algorithm based on color to improve computational efficiency, introduced the time–space model of multi-frame image sequences to improve the accuracy of traffic light detection, and combined these with support vector machines to identify traffic lights through the histogram feature of the oriented gradient of the image. The above methods can realize the detection of traffic lights to a certain extent; however, traditional algorithms still face great challenges in practice due to their poor generalization ability and limited detection speed.

The target detection algorithm based on deep learning was thus developed for the detection of traffic lights to overcome the limitations of traditional algorithms. The authors of [8] used the YOLO algorithm to detect traffic lights. By optimizing the loss function of the network, the number of network grid units was increased from 7 × 7 to 11 × 11, which significantly improved the model’s detection effect on traffic lights. In [9], the authors used the Faster R-CNN algorithm to effectively improve the detection speed of traffic lights, obtained regional suggestions and regional scores through the sliding window mechanism in the regional suggestion network, input them to the ROI pooling layer to obtain regional suggestion features, then input these to the fully connected layer to complete the identification of traffic lights. In [10], the authors used YOLOv2 for the detection of traffic lights. The final convolutional layer was removed from the network, and three convolutional layers with dimensions of 3 × 3 were added. In order to enable the identification of fine grain features, a passthrough layer was inserted behind the last convolutional layer. Multiple scales were used to train both the LISA and LARA traffic light data sets. The final area under the PR curve (AUC) value was as high as 90.49%. The authors of [11] improved the structure of the single-shot multibox detector (SSD) and used Inception-v3 to replace VGG-16 as the basic network. By combining background context information and local information to detect and recognize signal lights, a 95% detection rate was obtained with its self-made traffic light data sets. In [12], the authors combined a priori map information with the YOLOv3 algorithm to detect traffic lights. Ideal results were achieved by setting the default input size of the network to 608 × 608 and using multi-scale training. In [13], the authors improved the YOLOv3 algorithm. Multiple feature fusions were completed by combining with the up-sampling operation and the fused features were sent to the detection layer to achieve better detection results. The above target detection algorithm based on deep learning can realize the rapid detection of traffic lights under GPU acceleration, but the detection accuracy could not be guaranteed.

The YOLOv4 algorithm, which is a recent update in the YOLO series and has a better performance than many target detection algorithms, can be used to detect traffic lights [14]. As the most excellent target detection algorithm of the YOLO series, the YOLOV4 algorithm can realize the high-precision real-time detection of targets, but as with all target detection algorithms there is still a common problem—that is, it is not sensitive enough for small target detection. For example, the authors of [15] introduced the YOLOv4 target detection network to improve the detection accuracy and speed of the dial area, replaced Hough transform with image segmentation technology, and used an improved U-net network to improve the accuracy of pointer detection. At the same time, the automatic calibration of the dial was realized based on image segmentation. In [16], the authors introduced a method to obtain the best size of the region of interest (ROI) dynamically. Firstly, prior maps containing sufficient traffic light information were generated based on multi-sensor data. Then, by analyzing the relationship between the error of the sensors and the optimal size of the ROI, the adaptively dynamic adjustment (ADA) model was built. Furthermore, according to the multi-sensor data fusion positioning and ADA model, the optimal ROI could be obtained to predict the location of traffic lights. Finally, YOLOv4 was employed to extract and identify the image features. However, traffic lights, as small targets, were no longer sensitive to the positioning and color resolution due to the lack of shallow features as the network deepened [17].

In order to solve these problem and improve the detection accuracy of traffic signal lights, in this paper improvements of the YOLOv4 algorithm are investigated. The shallow features are fused with deep features after up-sampling to realize the enhancement of shallow features and improve the positioning and color resolution ability of the YOLOv4 algorithm for small targets. The Gaussian model is used to process the coordinates of the predicted bounding box and calculate the uncertainty of the bounding box in order to improve the reliability of the predicted bounding box and further improve the detection performance of the YOLOv4 algorithm for small targets.

## 2. Basics of YOLOv4 Algorithm

This section introduces the principle and network structure of the YOLOv4 algorithm.

### 2.1. The Principle of YOLOv4 Algorithm

The YOLOv4 algorithm divides the network input into *S* × *S* grid units; then, each grid unit predicts *B* bounding boxes, the bounding box confidence, and *C* category probabilities. The confidence of the predicted bounding box reflects whether the predicted bounding box contains objects and the accuracy of the position when the objects are included. The accuracy is expressed as the intersection over union (IOU) of the predicted bounding box and the real bounding box according to Equation (1).

(1) $$confidence\;=\;\mathrm{Pr}(\mathrm{object})\;\times \;{\mathrm{IOU}}_{pred}^{truth}$$

where *confidence* is the confidence of the bounding box and Pr(object) is the probability of the object being detected in the grid.

**Figure 1:**
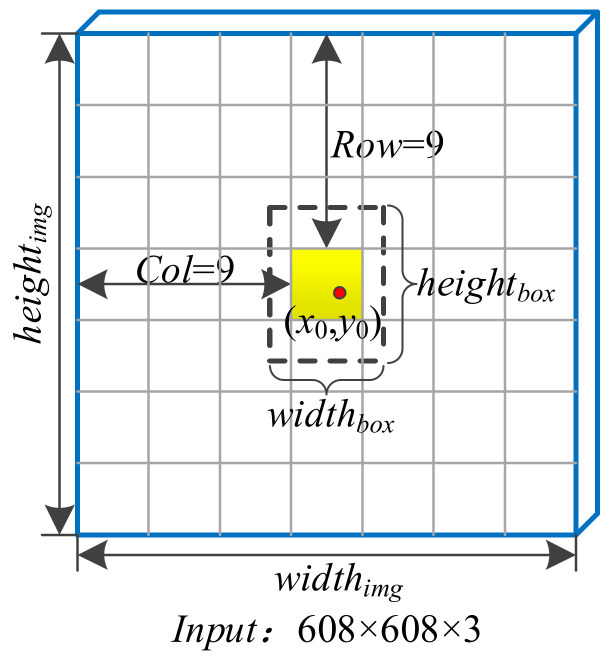
Normalization of predicted bounding box.

By setting the category confidence threshold, the bounding boxes with a category confidence higher than the threshold are screened out and the non-maximum suppression algorithm is used to obtain the final bounding box. The predicted bounding box includes the four parameters *t_x_*, *t_y_*, *t_w_*, and *t_h_*. In order to reduce the influence of singular samples on the network, the YOLOv4 algorithm normalizes the above parameters. As shown in Figure 1, the network input is an image with a size of 608 × 608. The input image is divided into 19 × 19 grid units. The width and height of the entire image are *width_img_* and *height_img_*, respectively, which are divided into s × s grid units. The dotted line is the predicted bounding box. The center point coordinates are (*x*_0_,*y*_0_) and the grid position of the center point is (*row*,*col*). The width and height of the bounding box are *width_box_* and *height_box_*, respectively. The normalization method is described as follows.

(1) Boundary box width and height normalization according to Equations (2) and (3), respectively.

(2) $$t_{w}\;=\;\frac{width_{box}}{width_{img}}$$

(3) $$t_{h}\;=\;\frac{height_{box}}{height_{img}}$$

(2) Center point coordinate normalization according to Equations (4) and (5).

(4) $$t_{x}\;=\;x_{0}\;\cdot \;\frac{s}{width_{img}}\;-\;col$$

(5) $$t_{y}\;=\;y_{0}\;\cdot \;\frac{s}{height_{img}}\;-\;row$$

### 2.2. CSPDarknet-53 Feature Extraction Network

**Figure 2:**
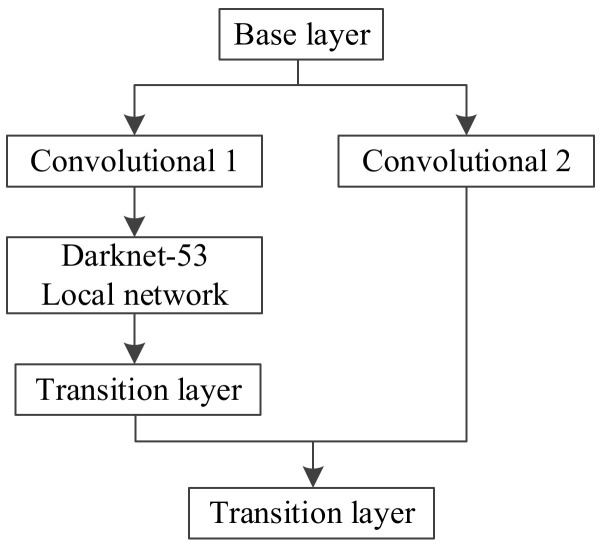
Cross-stage feature fusion strategy.

The CSPDarknet-53 feature extraction network is based on Darknet-53 and optimized by adding a cross-stage feature fusion strategy [18]. To prevent repeated gradient information from being obtained in different layers, the idea of splitting and fusion is introduced across stages to maximize the differences in gradient combinations. In the process of splitting and fusion, the gradient flow is truncated, meaning that the gradient information will not be reused and the generation of redundant information will be minimized. Applying the cross-stage feature fusion strategy to the local network of Darknet-53 can reduce the computational complexity of the feature extraction network and improve the speed and accuracy of the network’s reasoning. The cross-stage feature fusion strategy is shown in Figure 2.

**Figure 3:**
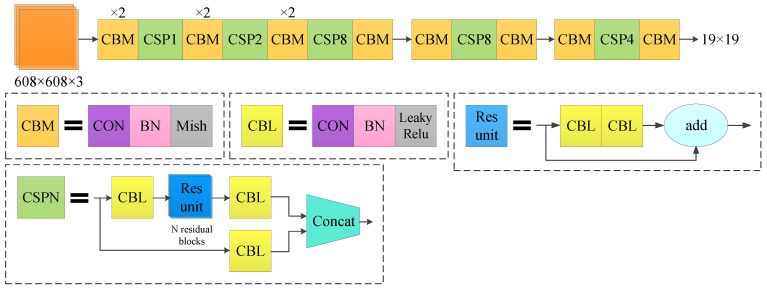
Network structure diagram of CSPDarknet-53.

CSPDarknet-53 retains the original 52 convolutional layers in Darknet-53 and adds a cross-stage feature fusion strategy to the Darknet-53 local network to effectively reduce the possibility of duplication in the information integration process. The network structure diagram is shown in Figure 3.

Compared with Darknet-53, CSPDarknet-53 can greatly reduce the amount of calculation necessary, increase the network inference speed, reduce the network memory consumption, and improve the network accuracy.

### 2.3. YOLOv4 Algorithm Loss Function

The loss function of the YOLOv4 algorithm is composed of three parts: the prediction error of the bounding box coordinates, the confidence error of the bounding box, and the category prediction error. The calculation formula of the loss function of the YOLOv4 algorithm is shown as Equation (6), and the meaning of each parameter of the loss function are shown in Table 1.

(6) $$\begin{array}{cc} Loss\;= & \lambda_{\mathrm{coord}}\overset{S^{2}}{\underset{i\;=\;0}{\Sigma }}\;\overset{B}{\underset{j\;=\;0}{\Sigma }}1_{ij}^{\mathrm{obj}}(2\;-\;w_{i}\;\times \;h_{i})[{\left(x_{i}\;-\;{\hat{x}}_{i}\right)}^{2}\;+\;{\left(y_{i}\;-\;{\hat{y}}_{i}\right)}^{2}]\;+\;\\ & \lambda_{\mathrm{coord}}\overset{S^{2}}{\underset{i\;=\;0}{\Sigma }}\;\overset{B}{\underset{j\;=\;0}{\Sigma }}1_{ij}^{\mathrm{obj}}(2\;-\;w_{i}\;\times \;h_{i})[{\left(w_{i}\;-\;{\hat{w}}_{i}\right)}^{2}\;+\;{\left(h_{i}\;-\;{\hat{h}}_{i}\right)}^{2}]\;-\;\\ & \overset{S^{2}}{\underset{i\;=\;0}{\Sigma }}\;\overset{B}{\underset{j\;=\;0}{\Sigma }}1_{ij}^{\mathrm{obj}}[{\hat{C}}_{i}\log(C_{i})\;+\;(1\;-\;{\hat{C}}_{i})\log(1\;-\;C_{i})]\;-\;\\ & \lambda_{\mathrm{noobj}}\overset{S^{2}}{\underset{i\;=\;0}{\Sigma }}\;\overset{B}{\underset{j\;=\;0}{\Sigma }}1_{ij}^{\mathrm{noobj}}[{\hat{C}}_{i}\log(C_{i})\;+\;(1\;-\;{\hat{C}}_{i})\log(1\;-\;C_{i})]\;-\;\\ & \overset{S^{2}}{\underset{i\;=\;0}{\Sigma }}1_{i}^{\mathrm{noobj}}\overset{S^{2}}{\underset{c\;\in \;\mathrm{classes}}{\Sigma }}[{\hat{p}}_{i}(c\log(p_{i}(c))\;+\;(1\;-\;{\hat{p}}_{i}(c))\log(1\;-\;p_{i}(c))] \end{array}$$

## 3. YOLOv4 Algorithm Network Improvement

In order to improve its ability to detect small targets, the YOLOv4 algorithm network needs to be improved. A method for shallow feature enhancement is proposed to improve the network’s ability to locate small targets, improve its color resolution, and enhance its sensitivity for small target detection. The bounding box uncertainty prediction mechanism is introduced to improve the reliability of predicting the bounding box.

### 3.1. YOLOv4 Algorithm Network Structure Improvement

The YOLOv4 algorithm uses the CSPdarknet-53 feature extraction network. As the network deepens, the receptive field increases while the dimension of the feature map decreases. Meanwhile, the features gradually become abstract and the semantic features become more and more obvious. Nevertheless, the location information becomes more and more fuzzy, which makes it impossible to achieve the precise detection of small targets. In view of this, a shallow feature enhancement method is proposed that combines shallow features with high-level semantic features to improve the precise positioning and recognition of small targets by the YOLOv4 algorithm.

The original feature extraction network of the YOLOv4 algorithm, CSPDarknet-53, uses a CSP strategy to ensure the feasibility of the shallow feature enhancement method and achieve the best results without destroying its network structure. This paper proposes two feature fusion improvement strategies—namely: fusing the 11th layer and 127th layer features while fusing the 23rd layer and 117th layer features after processing, and fusing the 23rd layer and 127th layer features while fusing the 54th layer and 117th layer features after processing. Two factors should be taken into consideration when realizing the strategies. Firstly, when the 11th layer shallow features are merged with the 127th layer features, the 127th layer features need to be upsampled 4 times before they can be merged with the 11th layer features. Secondly, the dimension of the generated features is as high as 304 × 304 × 255, and the large amount of additional calculations cannot achieve the real-time detection of signal lights.

On these grounds, the following methods are used to enhance the shallow features: (1) the high-level semantic features of the 117th layer are up-sampled; (2) the shallow features of the 54th layer are spliced; (3) the convolution operation is carried out; (4) the high-level semantic features of the 127th layer are up-sampled and the shallow features of the 23rd layer are spliced. After the convolution operation, the first-scale feature is obtained. The first-scale feature has dimensions of 152 × 152 × 255, the second-scale feature dimensions are 76 × 76 × 255, and the third-scale feature dimensions remain unchanged at 19 × 19 × 255. While ensuring the high detection accuracy for individual larger traffic lights, the detection accuracy of most small targets is improved. As the improved algorithm does not destroy the network structure of CSPDarknet-53, it ensures that some large traffic lights still have a high detection accuracy. At the same time, it integrates shallow features with more specific and obvious location information with the deep features, takes into account the advantages of shallow features and deep features, and improves the detection accuracy of traffic lights for most small targets.

**Figure 4:**
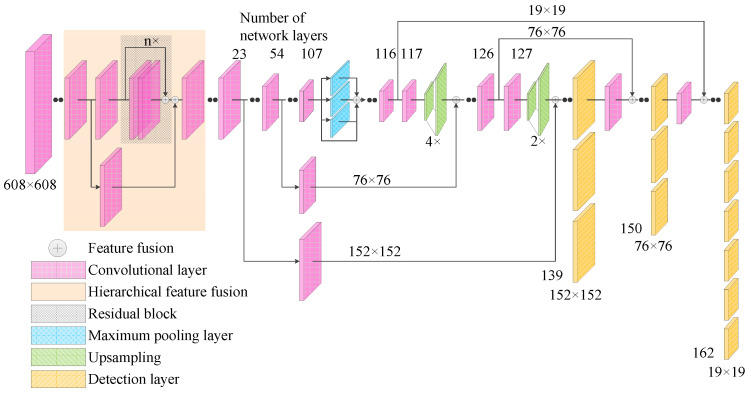
The network structure of the Improved YOLOv4 algorithm.

The network structure of the Improved YOLOv4 algorithm is shown in Figure 4. Firstly, the image input required by YOLOv4 algorithm is a multiple of 32, and the input traffic light data need to be processed. If the input size is set too small, the detection accuracy will be low. Experimental verification shows that if the input size is set above 608 × 608 × 3, the detection accuracy will not improve much, while the detection time will be slightly longer if the calculation is too large. Therefore, the traffic light data are scaled from the size of 1280 × 960 × 3 to the size of 608 × 608 × 3 in three channels as the input for the entire network. Secondly, 53-CSPDarket network is used for the feature extraction of input data, and input data with a size 3 × 3 and 1 × 1 are alternately used for convolution operation. In order to avoid the problem of the dimensions of feature graph decreasing with the deepening of the convolution depth, with the features gradually becoming abstracted and insensitive to small target detection, the above method is used to perform shallow feature fusion based on the original YOLOv4 algorithm. Finally, the three-scale characteristic information is formed and the detection and recognition of traffic lights are completed to improve the detection and recognition accuracy of traffic lights by the YOLOv4 algorithm.

### 3.2. Uncertainty Prediction of Bounding Box

The bounding box prediction of the original YOLOv4 algorithm only predicts the coordinate information, while the accuracy of the bounding box is not processed. Consequently, the accuracy of the predicted bounding box coordinates cannot be judged from the results [19,20]. In order to further strengthen the YOLOv4 algorithm’s ability to detect traffic lights, a bounding box uncertainty prediction mechanism is added to the YOLOv4 algorithm to predict the uncertainty of each coordinate information and improve the accuracy of the predicted bounding box.

In the original YOLOv4 algorithm, the coordinate information (*t_x_* and *t_y_*) of the center point of the bounding box and the size information (*t_w_* and *t_h_*) of the bounding box are extracted through bounding box regression. However, these parameters can only provide the position and size of the bounding box and cannot represent the reliability of the bounding box. In this paper, the uncertainty calculation is added to the calculation of the confidence level and a single Gaussian model of *t_x_*, *t_y_*, *t_w_*, and *t_h_* is used to model the uncertainty of the prediction frame. The Gaussian model is expressed by Equation (7).

(7) p(y|x) = N(y;μ(x),Σ(x))

where *μ*(*x*) represents the mean function and ∑(*x*) represents the variance function.

To predict the uncertainty of the bounding box, the coordinate information of the predicted bounding box is modeled as the mean and variance. The outputs of the bounding box are ${\hat{\mu }}_{t_{x}}$, ${\hat{\Sigma }}_{t_{x}}$, ${\hat{\mu }}_{t_{y}}$, ${\hat{\Sigma }}_{t_{y}}$, ${\hat{\mu }}_{t_{w}}$, ${\hat{\Sigma }}_{t_{w}}$, ${\hat{\mu }}_{t_{h}}$, and ${\hat{\Sigma }}_{t_{h}}$. Due to the detection layer structure in the network, the Gaussian parameters of *t_x_*, *t_y_*, *t_w_*, and *t_h_* are preprocessed using the Sigmoid function according to Equations (8)–(10).

(8) $$\mu_{t_{x}}\;=\;\sigma ({\hat{\mu }}_{t_{x}}),\;\;\mu_{t_{y}}\;=\;\sigma ({\hat{\mu }}_{t_{y}})\;\;\;\mu_{t_{w}}\;=\;{\hat{\mu }}_{t_{w}},\;\;\mu_{t_{h}}\;=\;{\hat{\mu }}_{t_{h}}$$

(9) $$\begin{array}{l} \Sigma_{t_{x}}\;=\;\sigma ({\hat{\Sigma }}_{t_{x}}),\;\;\Sigma_{t_{y}}\;=\;\sigma ({\hat{\Sigma }}_{t_{y}})\\ \Sigma_{t_{w}}\;=\;\sigma ({\hat{\Sigma }}_{t_{w}}),\;\;\Sigma_{t_{h}}\;=\;\sigma ({\hat{\Sigma }}_{t_{h}}) \end{array}$$

(10) $$\sigma (x)\;=\;\frac{1}{1\;+\;\exp(-x)}$$

The average value of each coordinate in the detection layer is the predicted coordinate of the bounding box, and each variance represents the uncertainty of its corresponding coordinate. In Equation (8), ${\hat{\mu }}_{t_{x}}$ and ${\hat{\mu }}_{t_{y}}$ represent the center coordinates of the bounding box in the grid, so the Sigmoid function is used to process it as a value between 0 and 1. $\mu_{t_{w}}$ and $\mu_{t_{h}}$ represent *t_w_* and *t_h_* in YOLOv4. Since their scale changes may exceed the grid size of the center point of the bounding box, the Sigmoid function is not used for processing. The variance of each coordinate information in Equation (9) is processed by the Sigmoid function to be between 0 and 1. In the Gaussian distribution, the greater the variance is, the greater the change in the distribution will be. Since each variance represents the uncertainty of its corresponding coordinate, the closer the processed variance is to 0, the smaller the uncertainty will be and the more reliable the predicted bounding box will be; the closer it is to 1, the greater the uncertainty will be and the less reliable the predicted bounding box will be.

The change in the calculation method of the bounding box confidence during prediction is calculated using Equation (11).

(11) $$confidence\;=\;\mathrm{Pr}(\mathrm{object})\;\times \;{\mathrm{IOU}}_{pred}^{truth}\;\times \;(1\;-\;Uncertainty_{aver})$$

where *Uncertainty_aver_* is the mean value of the uncertainty for each coordinate’s information.

Compared with the large target, if a bounding box of the same size is used in the detection of the small target, the variance will be large due to the small target occupying fewer pixels, resulting in a low confidence of the bounding box, and the bounding box can easily be abandoned. In small target detection, the variance of the bounding box with a smaller size is smaller and the mean value obtained for the uncertainty will be smaller, leading to the higher confidence of the predicted bounding box. By setting the category confidence threshold, the bounding boxes with a category confidence higher than the threshold are screened out, and the redundant prediction boundary boxes are removed by a non-maximum suppression algorithm to obtain the final boundary boxes. In other words, by adding the bounding box uncertainty prediction mechanism to the YOLOv4 algorithm, the predicted bounding box with smaller parameters becomes more suitable for small target detection tasks.

### 3.3. Performance Analysis of Improved YOLOv4 Algorithm for Small Target Detection

The pixel size of the pictures in the VOC2007 data set varies, generally being 500 × 375 pixels (horizontal image) or 375 × 500 pixels (vertical image), and the width and height cannot deviate by more than 100 pixels. In order to verify the effectiveness of the improved algorithm for the problem of small target detection, this paper selects small targets in the VOC2007 data set whose width and height are less than one tenth that of the original image (that is, a target that occupies 50 × 37 pixels or 37 × 50 pixels, and the width and height deviation does not exceed 10 pixels) for experimental verification. There are eight types of small targets—namely, airplanes, birds, boats, bottles, cars, dogs, sheep, and people—with a total of 1164 tags.

The YOLOv4 algorithm that only increases the reliability of Gaussian model calculation coordinates is named YOLOv4-v1, and the YOLOv4 algorithm that only increases the shallow feature enhancement mechanism is named YOLOv4-v2. The YOLOv4 algorithm optimized by using two improved methods is named Improved YOLOv4. On the VOC2007 small target data set, experiments were carried out using the YOLOv4-v1, YOLOv4-v2, and Improved-YOLOv4 algorithms.

It can be seen from Table 2 that the Improved YOLOv4 algorithm has a significantly improved detection accuracy for small targets. By using two methods simultaneously, the detection accuracy of the Improved YOLOv4 algorithm is 8.08% higher than the mean average precision (mAP) value of the original YOLOv4 algorithm. It can be noticed that the overall accuracy and recall rate of the Improved YOLOv4 algorithm are also significantly improved. Although the detection speed of the Improved YOLOv4 algorithm using the shallow feature fusion method has slowed down, it can still achieve the real-time detection of the target.

## 4. Experiment and Analysis

### 4.1. Experimental Platform and Data

The operating system of the experimental platform was Ubuntu 16.04 with the processor Inter(R)-CPU-E5-2620-v4. In order to improve the computing speed and reduce the training time, an Nvidia GeForce GTX 1080Ti graphics card, was used in the Darknet framework. CUDA 8.0, and cuDNN 6.0 used GPU for acceleration.

In order to verify the performance of the Improved YOLOv4 algorithm for traffic signal detection, experiments were carried out using the LISA traffic light data set of the Intelligent and Safe Automobile Laboratory of the University of California, San Diego [21]. The Bumblebee XB3 camera was installed in the central position at the top of the vehicle to shoot the traffic signal lights during the sample collection for this data set. The large amount of data collected included various scenes, such as strong illumination, target coverage, and night. These scenes raised the difficulty of the traffic signal light identification and were able to better verified the robustness of the algorithm. The dataset was equipped with complete labels; it is summarized in Table 3, where LISA-dayTrain is the training set and LISA-daySeq1 is the test set.

In addition, in order to verify the scalability and generalization of the improved YOLOv4 algorithm, experiments were carried out using the LaRa dataset collected on the streets of Paris, France. The samples of this data set were collected by the Marling F-046C camera sensor. The camera was mounted behind the interior rear-view mirror, and the vehicle speed was less than 50 km/h. The dataset is summarized in Table 4. In order to facilitate the experiment, 449 samples labelled as “ambiguous” were removed from the 9168 labelled samples, and the remaining 8719 samples were used to divide the training set and the test set. Among these, the training set contained 7051 samples and the test set contained 1668 samples.

### 4.2. YOLOv4 Algorithm Anchor Parameter Calculation

The YOLOv4 algorithm extends the anchor mechanism. Anchor is a set of a priori candidate frames with a fixed aspect ratio to constrain the range of the predicted object. In the training process of the network, through the pre-set anchor parameters the size of the predicted bounding box is continuously adjusted, and the optimal predicted bounding box is gradually determined. Therefore, when training traffic signal data, it is very important to set appropriate network parameters according to the characteristics of the traffic signal data. In order to filter the anchor parameters that are most suitable for traffic lights, in this paper we used the K-means++ clustering algorithm to cluster the traffic light data, calculate the similarity of the input samples, and obtain the anchor parameters suitable for the traffic light data.

**Figure 5:**
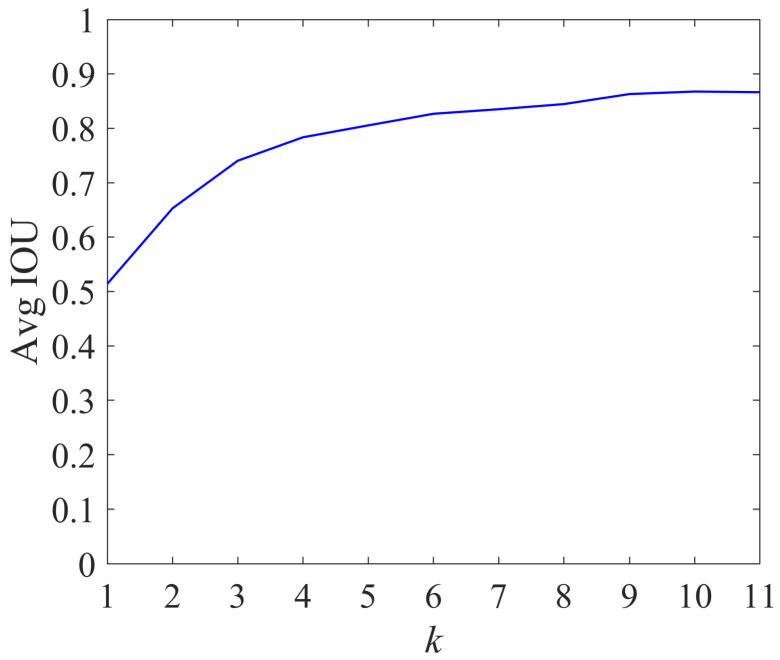
The change curve of Avg IOU.

We selected the number of different clusters *k* and used the K-means++ algorithm to cluster the traffic light data. The average intersection over union (AVG IOU) of the real box and the predicted box varied with the value of *k*, as shown in Figure 5.

It can be seen from Figure 5 that as the number of clusters *k* increased, the average intersection over union tended to become more flat. The larger the value of *k* was, the smaller the gap between the real box and the predicted box was, which helped reduce the matching error in the training process. Therefore, in this paper we chose to use the a priori aspect ratio when *k* = 9 as the anchor parameter of the Improved YOLOv4 algorithm; the a priori aspect ratios corresponding to different *k* values are shown in Table 5.

### 4.3. Model Training Analysis

**Figure 6:**
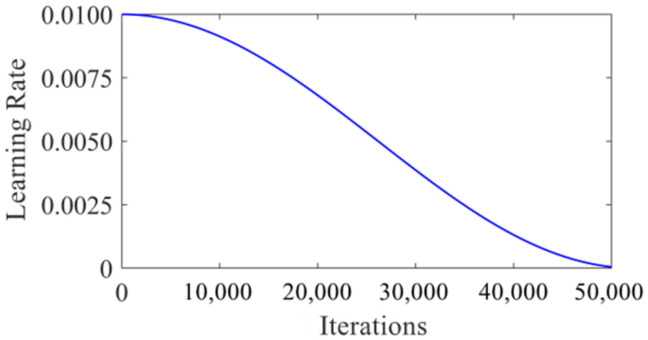
Attenuation curve of the cosine function.

In all experiments, the K-means++ clustering algorithm was used to calculate the anchor parameters corresponding to the traffic light data set and replace the original anchor parameters used in the algorithm. The maximum number of iterations was set as 50,000, and the initial learning rate was set as 0.01. In order to prevent the gradient explosion from occurring when the learning rate was too high in the training process, the cosine function attenuation method [22] was used to attenuate the learning rate. The attenuation curve of the cosine function is shown in Figure 6.

**Figure 7:**
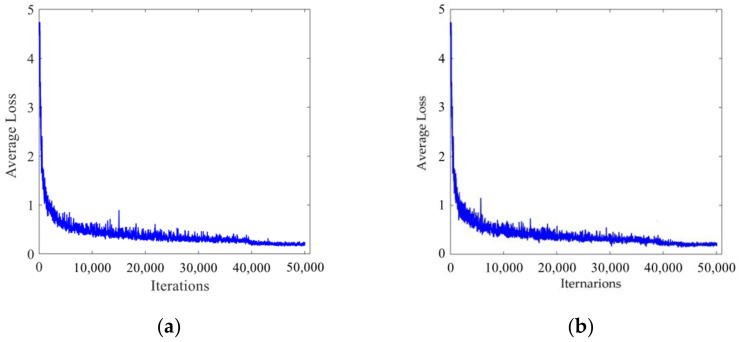
Relation curve of average loss and the number of iterations. (**a**) LISA traffic light data set. (**b**) LaRa traffic light data set.

During the training process, all training parameters of the network were recorded. Figure 7 shows the curve of average loss changing with the number of iterations. It can be seen from the figure that the average loss in the initial training stage was relatively large. As the number of iterations increased, the average loss decreased continuously and finally stabilized, achieving an ideal training effect.

**Figure 8:**
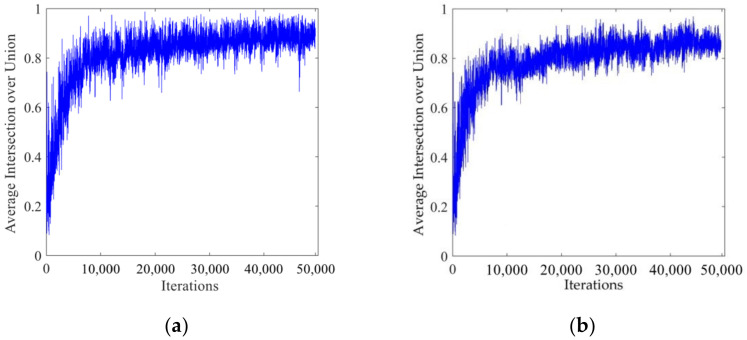
Relation curve of the average intersection over union and the number of iterations. (**a**) LISA traffic light data set. (**b**) LaRa traffic light data set.

Figure 8 shows the curve of the average intersection over union with the number of iterations. It can be seen from the figure that the average intersection over union was very small at the beginning of the training. As the number of iterations increased, the average intersection over union gradually increased. After the number of iterations reached 45,000, the average intersection over union could be maintained at about 0.9 to achieve the ideal training effect.

### 4.4. Analysis of Traffic Lights Detection Performance

In the traffic light detection, the problem was that the traffic light only occupied a small number of effective pixels. The background of traffic lights in natural scenes is complex and changeable. Strong light, evening, and weather conditions will affect the detection effect of traffic lights. In order to verify the effectiveness of the algorithm proposed in this paper for small target traffic light detection within a complex background, the AUC value specified in the Vision for Intelligent Vehicles and Applications (VIVA) Challenge Competition was used as the evaluation index for this section of the experiment, and the intersection over union of true positive samples in the calculation should be greater than 0.5.

**Figure 9:**
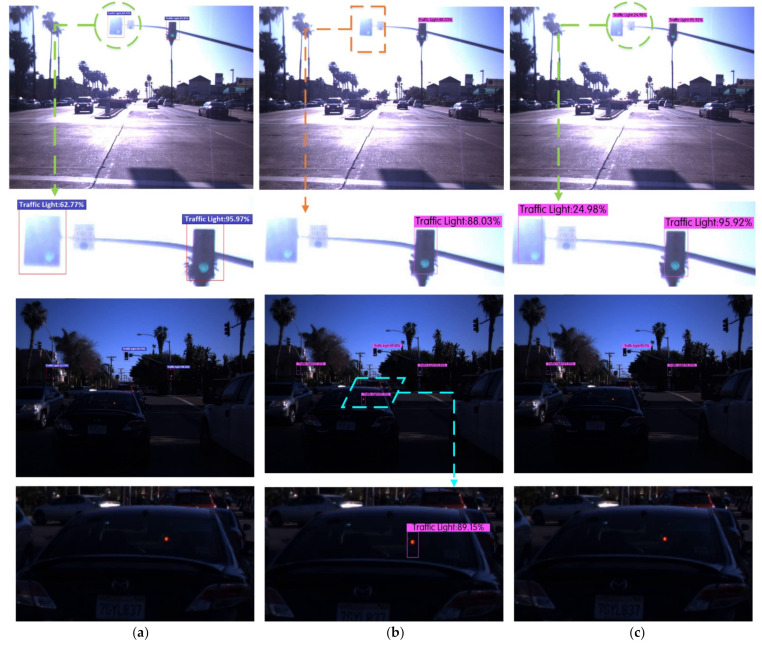

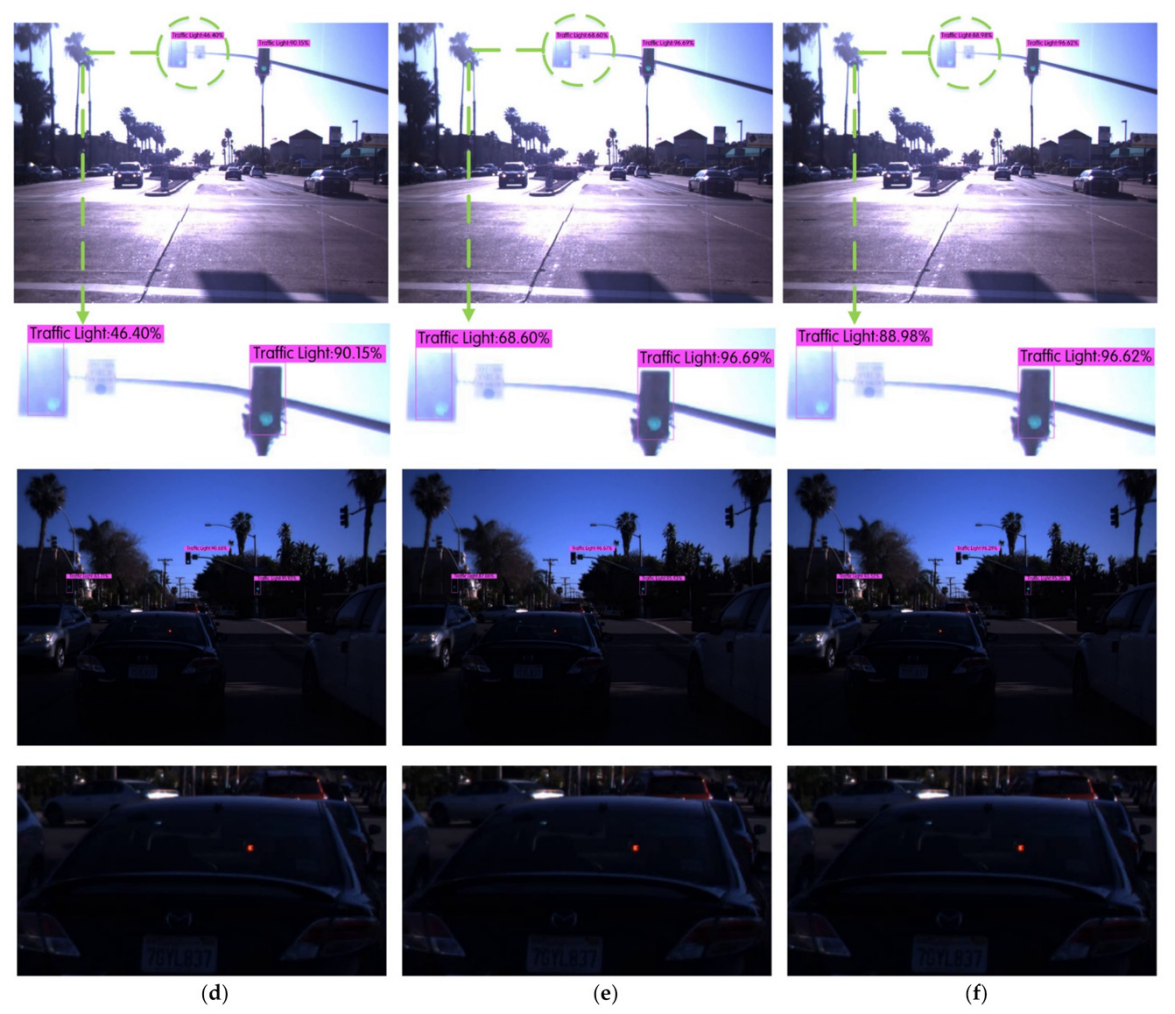
Comparison of detection results using multiple algorithms for traffic lights in different scenarios in the LISA data set. (**a**) Faster R-CNN. (**b**) YOLOv3. (**c**) YOLOv4. (**d**) YOLOv4-v1. (**e**) YOLOv4-v2. (**f**) Improved YOLOv4.

We detected traffic lights according to the requirements of the VIVA Challenge Competition, where the category name was Traffic Light. Six experiments were performed on the LISA traffic light dataset using the Faster R-CNN, YOLOv3, YOLOv4, YOLOV4-V1, YOLOV4-V2, and Improved YOLOv4 algorithms. After full training, the detection results of the various traffic signal lights in different scenes were compared, as shown in Figure 9. The yellow rectangle represents the missing spot, the blue parallelogram represents the wrong spot, and the green circular represents the correct spot.

Figure 9 lists the detection effect maps and the local area enlarged images of traffic lights under different algorithms in the two scenarios of strong illumination and evening. It can be seen from the figure that the YOLOv3 algorithm missed targets and led to the false detection of traffic lights in strong light and in the evening, while the Faster R-CNN, YOLOv4, YOLOv4-v1, YOLOv4-v2, and Improved YOLOv4 algorithms avoided missed and false detections in the legend.

By testing 4060 traffic light data points in the LISA-daySeq1 test set, the Improved YOLOv4 algorithm was able to greatly reduce the missed and false detection of traffic lights and the detection accuracy was significantly improved. The performance indicators of the traffic lights detection are shown in Table 6. The results show that although the Faster R-CNN algorithm had a high AUC value, the detection time was longer, meaning that it could not achieve the real-time detection of traffic lights. Compared with the original YOLOv4-v1 algorithm, the AUC value, precision, and recall rate of YOLOv4-v2 algorithm were significantly improved. The Improved YOLOv4 algorithm had the highest AUC value, precision rate, and recall rate, which were much higher than those of the original YOLOv3 algorithm, indicating that the two improved methods proposed in this paper were suitable for traffic light detection. Since the shallow feature enhancement mechanism was used in the YOLOv4-v2 and Improved YOLOv4 algorithms, which increased the amount of network calculations, the detection speed was lower than that of the original YOLOv4 algorithm, but the average detection time of a single image of the Improved YOLOv4 algorithm was only 33.74 ms. Compared with the 101.48 ms taken by the Faster R-CNN algorithm, the Improved YOLOv4 algorithm had a higher detection accuracy while still achieving the real-time detection of traffic lights.

**Figure 10:**
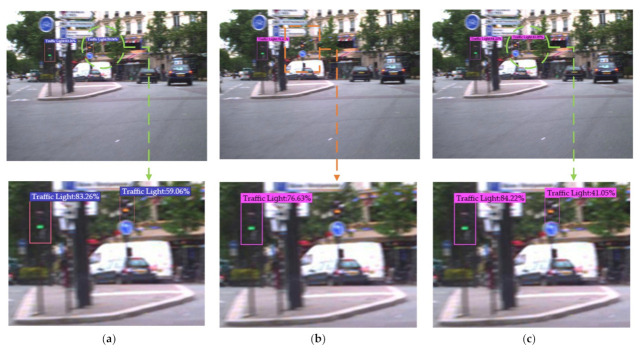

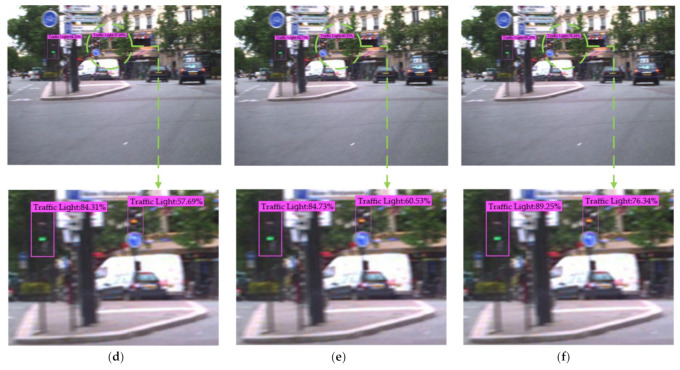
Comparison of the detection results using multiple algorithms for traffic lights in different scenarios in the LaRa data set. (**a**) Faster R-CNN. (**b**) YOLOv3. (**c**) YOLOv4. (**d**) YOLOv4-v1. (**e**) YOLOv4-v2. (**f**) Improved YOLOv4.

In order to further evaluate the performance of the Improved YOLOv4 algorithm for traffic light detection and verify the generalization of the algorithm, the above traffic light detection experiments were carried out again with the LaRa data set. After full training, the comparison of the detection results of different algorithms is shown in Figure 10. The yellow rectangle represents the missing spot, the blue parallelogram represents the wrong spot, and the green circular represents the correct spot.

By testing 1668 samples, the results further showed that the Improved YOLOv4 algorithm could greatly reduce the missed and false detection of traffic lights, and the detection accuracy was significantly improved. The performance indicators for traffic light detection are shown in Table 7. At the same time, by analyzing the detection results of the same data set in different scenarios and different data set conditions, this also fully proved that the Improved YOLOv4 algorithm proposed in this paper had generalization potential and scalability.

### 4.5. Analysis of Traffic Lights Recognition Performance

In order to verify the effectiveness and robustness of the Improved YOLOv4 algorithm for traffic light recognition, the traffic light recognition experiment in this section divided the green, red, and yellow traffic lights into Go, Stop, and Warning and adopted the evaluation index mAP that is commonly used in target detection algorithms as the experimental evaluation index.

**Figure 11:**
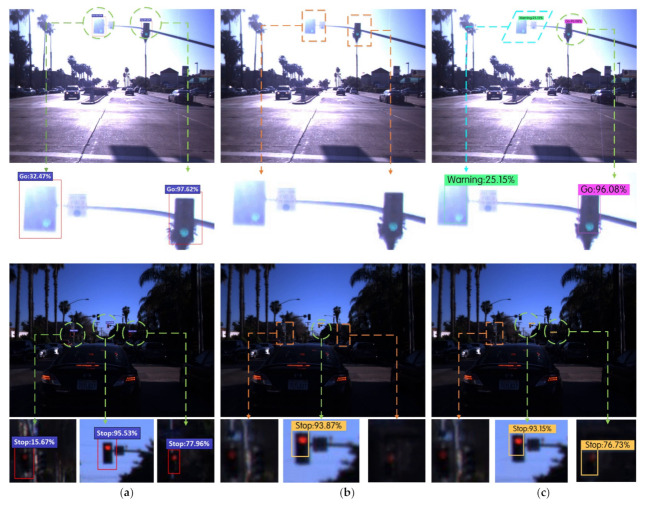

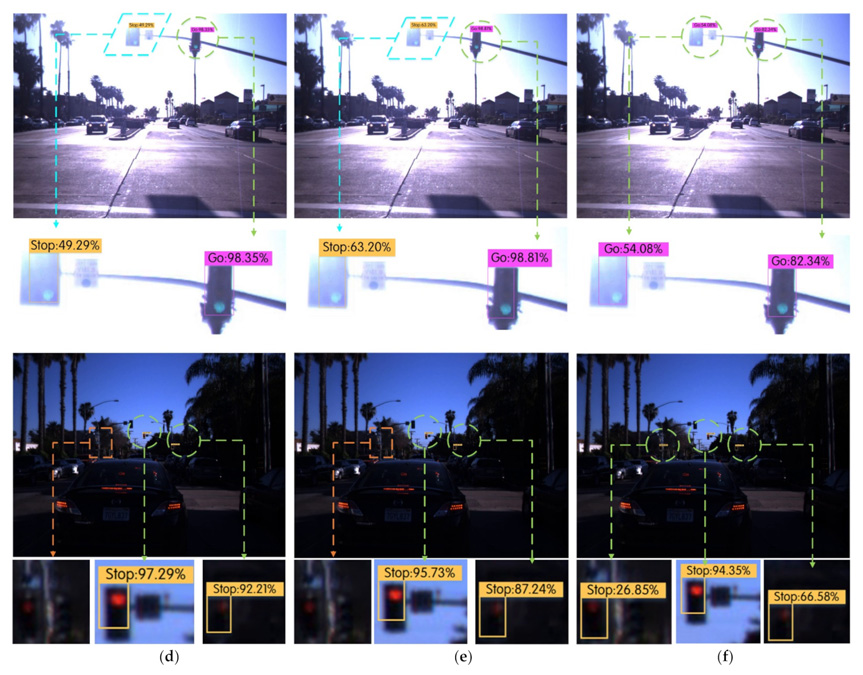
Comparison of recognition results using multiple algorithms for traffic lights in different scenarios in the LISA data set. (**a**) Faster R-CNN. (**b**) YOLOv3. (**c**) YOLOv4. (**d**) YOLOv4-v1. (**e**) YOLOv4-v2. (**f**) Improved YOLOv4.

The Faster R-CNN, YOLOv3, YOLOv4, YOLOv4-v1, YOLOv4-v2, and Improved YOLOv4 algorithms were used to carry out six groups of experiments on the LISA traffic light data sets. The detection results of various traffic signal lights in different scenes were compared, as shown in Figure 11, where the yellow rectangle represents the missing spot, the blue parallelogram represents the wrong spot, and the green circular represents the correct spot.

Figure 11 lists the detection effect maps and local area enlarged images of traffic lights under different algorithms in the two scenarios of strong illumination and evening. There were two cases of green traffic lights in the figure under strong light. The YOLOv3 algorithm missed detection, and the YOLOv4, YOLOv4-v1, and YOLOv4-v2 algorithms all had one case of false detection and one case of correct detection. The Faster R-CNN and Improved YOLOv4 algorithms could correctly identify all the green traffic lights in the figure, and there were three cases of red traffic lights in the figure in the evening. The YOLOv3 algorithm correctly identified only one obvious red traffic light, while the YOLOv4, YOLOv4-v1, and YOLOv4-v2 algorithms correctly identified two red traffic lights. The Faster R-CNN and Improved YOLOv4 algorithms correctly identified all red traffic signal lights in the figure. After testing on 4060 images in the test set, the Improved YOLOv4 algorithm could effectively reduce the occurrence of missed and false detections of traffic lights, and the recognition accuracy of traffic lights was significantly improved.

The performance indicators of traffic light identification are shown in Table 8. In the identification experiments of the YOLOv4-v1 and YOLOv4-v2 algorithms, their mAP values were significantly improved compared with the original YOLOv4 algorithm, which proved that the proposed two improved methods of shallow feature enhancement and boundary box uncertainty prediction could effectively improve the identification accuracy of the YOLOv4 algorithm for traffic lights.

**Figure 12:**
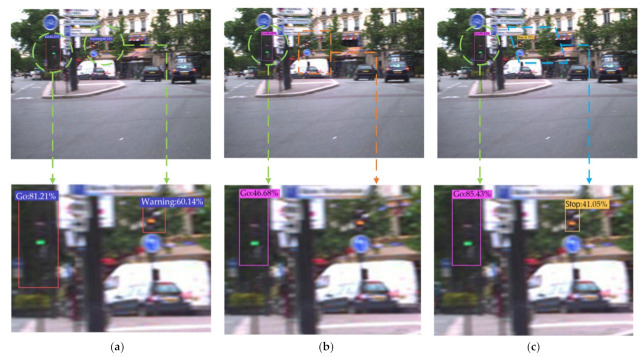

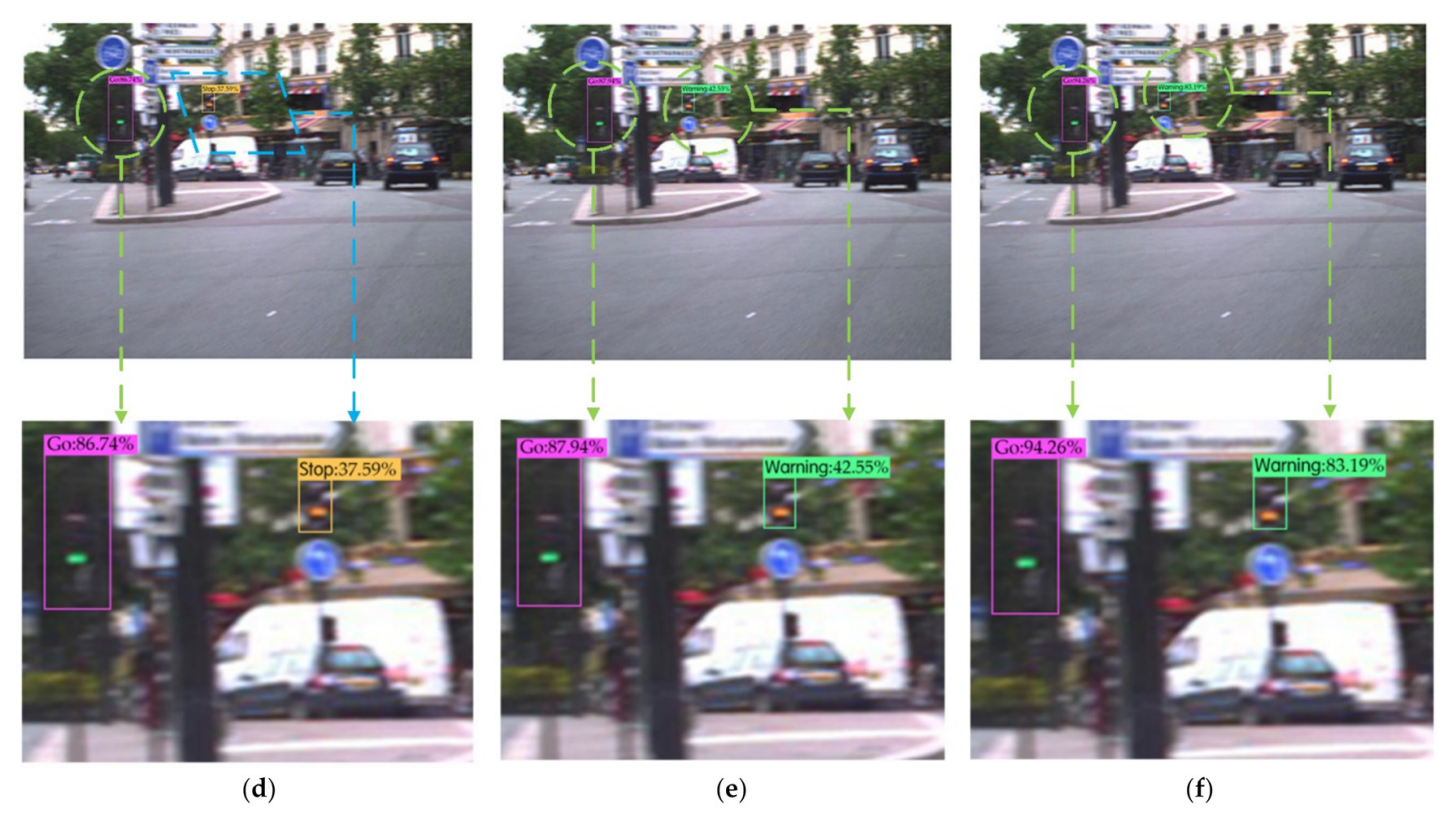
Comparison of recognition results using multiple algorithms for traffic lights in different scenarios in the LaRa data set. (**a**) Faster R-CNN. (**b**) YOLOv3. (**c**) YOLOv4. (**d**) YOLOv4-v1. (**e**) YOLOv4-v2. (**f**) Improved YOLOv4.

In order to further evaluate the performance of the Improved YOLOv4 algorithm for traffic light recognition, six groups of experiments were carried out on the LaRa data set using the above algorithms. The comparison of recognition results is shown in Figure 12, where the yellow rectangle represents the missing spot, the blue parallelogram represents the wrong spot, and the green circular represents the correct spot.

The performance indicators of traffic light identification are shown in Table 9. In the recognition experiments, the mAP value of the YOLOv4-v1 algorithm increased by 0.82% compared to the original YOLOv4 algorithm and the mAP value of the YOLOv4-v2 algorithm increased by 1.47% compared to the original YOLOv4 algorithm. These results further proved that the proposed two improved methods of shallow feature enhancement and boundary box uncertainty prediction could effectively improve the identification accuracy of traffic lights by the YOLOv4 algorithm, and these had a good scalability.

## 5. Conclusions

We proposed the use of the Improved YOLOv4 algorithm for traffic light detection and recognition. This method involved adding a shallow feature enhancement mechanism and a bounding box uncertainty prediction mechanism. By using the Improved YOLOv4 algorithm, the problem that the YOLOv4 algorithm was not sensitive to small targets was effectively solved and the accuracy of the traffic light detection and recognition was greatly improved. The experimental analysis was performed with the LISA traffic light data set and the LaRa traffic light data set, and the following conclusions were obtained.

(1) A shallow feature enhancement mechanism was applied to optimize the YOLOv4 algorithm. The accuracy of traffic light detection and recognition was effectively improved. For the two data sets of LISA and LaRa, the AUC is increased to 97.03% and 95.31% in the traffic signal detection experiments, respectively, while mAP is increased to 81.34% and 78.88% in the recognition experiments, respectively. Due to the increase in the number of network calculations, the detection time was increased to 33.99 ms and 39.63 ms, respectively. The results show that the method can effectively improve the detection and recognition accuracy of traffic lights. Although the amount of network calculation necessary was increased slightly, the method could still realize the real-time detection and recognition of traffic lights.
(2) A bounding box uncertainty prediction mechanism was applied to optimize the YOLOv4 algorithm, which effectively improved the accuracy of the YOLOv4 algorithm in the detection and recognition of traffic lights. The accuracy of traffic light detection and recognition was effectively improved. For the two data sets of LISA and LaRa, the AUC was increased to 96.84% and 94.73% in the traffic signal detection experiments and the mAP was increased to 79.93% and 78.23% in the recognition experiments. The detection time was reduced to 27.59 ms and 33.45 ms, respectively. The results show that, compared with the improved YOLOv4 algorithm, this method had little difference in detection and recognition speed but effectively improved the accuracy of the YOLOv4 algorithm in the detection and recognition of traffic lights.
(3) The Improved YOLOv4 algorithm involved using the two optimization methods, the shallow feature enhancement mechanism, and the bounding box uncertainty prediction mechanism. For the two data sets of LISA and LaRa, the AUC was increased by 1% and 1.19% in the traffic light detection experiments compared with the original YOLOv4 algorithm, respectively. In the recognition experiments, the mAP was improved by 2.86% and 2.56% compared with the original YOLOv4 algorithm, respectively. The robustness of the Improved YOLOv4 algorithm was evidenced by the significant reduction in the missed and false detection cases under complex traffic signal light backgrounds, such as strong illumination, target blocking, and evening. Additionally, when the improved YOLOv4 algorithm was tested on data sets collected by different cameras, the AUC and mAP are improved, which also proved the scalability of the algorithm. Although the calculation cost increased due to the increase in the number of network calculations, the increased detection time was only at the ms level, which could also ensure the real-time detection of traffic lights. This shows that the method described in this paper is a feasible method for use in real scenarios.

Prospective investigations: It is still a challenge to avoid missed and false detections of traffic lights due to the complexity and changeability of the background in traffic light detection scenarios. Considering the effectiveness of the Improved YOLOv4 algorithm in reducing the number missed and false detection cases, the target tracking should be focused on the identified traffic light to predict the movement trajectory and status of the traffic light relative to the vehicle in order to further improve the reliability of the Improved YOLOv4 algorithm for the detection and recognition of traffic lights.

## Figures and Tables

**Table 1:** The meaning of each parameter of the loss function.

Parameter	Meaning
*λ*_coord_	Coordinate loss weight
*λ*_noobj_	Does not include the target’s loss weight
$1_{ij}^{\mathrm{obj}}$	Whether the *j* anchor box of the *i* grid has targets
$1_{ij}^{\mathrm{noobj}}$	Whether the *j* anchor box of the *i* grid has no target
*S*	The number of input images in the grid cell division
*B*	The number of each grid cell projection boundary box
*C_i_*	Confidence
*p*	Category
*c* = 0,1,...,*C*	Category number
*i* = 0,1,...,*S*^2^	Grid unit number
*j* = 0,1,...,*B*	Bounding box number
$x_{i},y_{i},w_{i},h_{i}$	The abscissa, ordinate, width, and height of the center point of the prediction box
${\hat{x}}_{i},{\hat{y}}_{i},{\hat{w}}_{i},{\hat{h}}_{i}$	The abscissa, ordinate, width, and height of the center point of the real box

**Table 2:** Small target detection performance index of the VOC2007 data set.

Algorithm	AP/%								mAP/%	Precision/%	Recall/%	Detection Speed/ms
	Aeroplane	Bird	Boat	Bottle	Car	Dog	Person	Sheep				
YOLOv4	78.23	63.65	59.47	57.26	84.31	75.21	81.47	65.84	70.68	74.31	76.62	22.28
YOLOv4-v1	80.09	65.77	62.78	59.43	89.45	75.47	84.82	68.71	73.32	75.26	77.53	22.04
YOLOv4-v2	83.42	68.75	64.96	62.77	88.26	78.53	84.21	72.31	75.40	79.87	83.79	26.27
improved-YOLOv4	85.61	75.63	70.02	66.57	87.67	85.89	83.23	75.53	78.76	81.27	85.11	25.93

**Table 3:** Overview of the LISA traffic light data set.

Sequence Name	Number of Images	Number of Tags	Image Size
LISA-dayTrain	14,025	40,764	1280 × 960
LISA-daySeq2	6894	11,144	1280 × 960
LISA-daySeq1	4060	10,308	1280 × 960

**Table 4:** Overview of the LaRa traffic light data set.

Sequence Name	Number of Images	Number of Tags	Image Size
Lara_UrbanSeq1_JPG	11,179	9168	640 × 480

**Table 5:** A priori aspect ratio corresponding to different *k* values.

*k = 6*	*k = 7*	*k = 8*	*k = 9*	*k = 10*	*k = 11*
(6,13)	(7,14)	(6,12)	(6,13)	(6,13)	(6,13)
(8,16)	(10,21)	(7,17)	(8,16)	(8,16)	(8,16)
(10,24)	(12,28)	(9,17)	(10,24)	(10,24)	(10,23)
(13,28)	(16,34)	(10,24)	(13,27)	(13,27)	(13,24)
(18,40)	(18,44)	(13,28)	(16,34)	(15,34)	(13,30)
(26,53)	(25,42)	(16,37)	(18,44)	(20,39)	(17,32)
-	(27,58)	(21,44)	(25,41)	(18,47)	(17,41)
-	-	(27,58)	(23,55)	(25,42)	(25,41)
-	-	-	(29,59)	(23,54)	(22,52)
-	-	-	-	(29,59)	(28,55)
-	-	-	-	-	(30,70)

**Table 6:** Traffic light detection performance index in the LISA data set.

Algorithm	AUC/%	Precision/%	Recall/%	Detection Speed/ms
ACF	40.17	-	-	-
YOLOv2	90.49	-	-	-
Faster R-CNN	97.01	98.25	95.93	101.48
YOLOv3	92.32	93.03	92.97	24.38
YOLOv4	96.58	96.86	95.62	28.33
YOLOv4-v1	96.84	97.41	96.13	27.59
YOLOv4-v2	97.03	97.96	96.17	33.99
Improved YOLOv4	97.58	98.74	96.81	33.74

**Table 7:** Traffic light detection performance index in the LaRa data set.

Algorithm	AUC/%	Precision/%	Recall/%	Detection Speed/ms
ACF	39.28	-	-	-
YOLOv2	88.33	-	-	-
Faster R-CNN	94.92	96.63	93.74	163.80
YOLOv3	89.71	90.13	90.01	28.21
YOLOv4	94.66	95.03	94.67	32.39
YOLOv4-v1	94.73	95.02	94.40	33.45
YOLOv4-v2	95.31	96.76	95.61	39.63
Improved YOLOv4	95.85	97.98	95.77	40.17

**Table 8:** Traffic light recognition performance index in the LISA data set.

Algorithm	AP/%			mAP/%	Precision/%	Recall/%	Detection Speed/ms
	Go	Stop	Warning				
Faster R-CNN	73.29	91.63	78.97	81.29	82.18	83.79	101.48
YOLOv3	63.28	85.02	74.71	74.33	75.17	80.30	24.38
YOLOv4	71.46	89.97	76.43	79.29	80.17	81.99	28.33
YOLOv4-v1	73.02	90.23	76.54	79.93	81.42	82.97	27.59
YOLOv4-v2	73.93	91.86	78.25	81.34	82.07	83.58	33.99
Improved YOLOv4	76.67	91.26	78.53	82.15	83.59	84.85	33.74

**Table 9:** Traffic light recognition performance index in the LaRa data set.

Algorithm	AP/%			mAP/%	Precision/%	Recall/%	Detection Speed/ms
	Go	Stop	Warning				
Faster R-CNN	70.51	87.31	74.23	77.35	79.26	80.92	163.80
YOLOv3	60.59	81.29	73.42	71.76	72.52	77.65	28.21
YOLOv4	69.72	86.38	76.11	77.41	78.39	79.24	32.39
YOLOv4-v1	72.34	87.99	74.35	78.23	78.74	80.06	33.45
YOLOv4-v2	73.45	88.15	75.04	78.88	79.11	80.61	39.63
Improved YOLOv4	74.74	88.68	76.49	79.97	81.02	82.17	40.17

## Data Availability

Not applicable.
